# MRI features and prognostic evaluation in patients with subventricular zone-contacting IDH-wild-type glioblastoma

**DOI:** 10.2478/raon-2025-0029

**Published:** 2025-05-30

**Authors:** Shijiao Pan, Yang Chen, Shan Zhao, Jingjing Pan, Shengsheng Xu

**Affiliations:** Department of Radiology, the First Affiliated Hospital of Chongqing Medical University, Chongqing, China; Wuhan University of Science and Technology Medical College, Wuhan, China

**Keywords:** glioblastoma, subventricular zone, overall survival, MRI feature, prognosis

## Abstract

**Background:**

The subventricular zone (SVZ), the brain’s largest neural stem cells reservoir, plays a critical role in glioblastoma development and progression. This study aims to investigate the association between MRI features and SVZ contact in IDH-wild-type glioblastoma, as well as their prognostic significance to guide personalized diagnosis and treatment.

**Patients and methods:**

We retrospectively analyzed the MRI and clinical data of 371 patients with IDH-wild-type glioblastoma from The Cancer Imaging Archive. Tumors were classified into SVZ contact and non-contact group based on the spatial relationships between contrast-enhanced lesions and the SVZ on T1C imaging. Group differences were analyzed, and survival outcomes were assessed using Cox regression and Kaplan-Meier analyses.

**Results:**

SVZ contact was observed in 64.4% of patients, these patients exhibited significantly shorter overall survival (OS) compared to the SVZ non-contact group (11.0 *vs*. 17.5 months, p < 0.001), larger tumor size (5.07 *vs*. 3.31 cm, p < 0.001), and higher rates of crossing the midline (11.7% *vs*. 0%, p < 0.001). They also showed higher rates of cystic lesions and necrosis. Cox regression confirmed SVZ contact as an independent predictor of poor OS (p = 0.027), alongside multifocal lesions and age. OS significantly differed by SVZ contact regions (p < 0.001), with temporal horn contact linked to longer OS and body contact to shorter OS.

**Conclusions:**

SVZ contact is an independent prognostic factor for OS in IDH-wild-type glioblastoma, they exhibit larger tumor size, higher rates of crossing the midline, and multifocality. Prognostic differences among SVZ contact regions warrant further investigation to explore the role of their distinct microenvironments.

## Introduction

Glioblastoma (GBM) is the most common malignant primary brain tumor in adults, accounting for approximately 49.1% of all malignant brain tumors.^1^ It is characterized by high rates of disability, recurrence, and significant clinicopathological heterogeneity. The current standard-of-care for GBM involves surgical resection, followed by postoperative radiotherapy and adjuvant temozolomide chemotherapy.^2^ Despite aggressive treatment, patient prognosis has not improved significantly, with a five-year survival rate of only 6.8%.^3^

In recent years, to improve the prognosis of patients with GBM, many researchers have paid attention to the cell origin of GBM. Although there is no consensus on the cellular origin of GBM, most scholars believe that neural stem cells (NSCs) in the subventricular zone (SVZ) may be the main cell of origin of GBM.^4,5^ The SVZ is generally defined as the 3–5 mm lateral border of the lateral ventricles, which harbors the largest reservoir of NSCs in the adult brain. Due to its extensive contact with cerebrospinal fluid (CSF), the enrichment of various growth factors in the CSF may further enhance the malignant potential of tumor cells.^6,7^ Previous studies have suggested that the CXCL12/CXCR4 signaling pathway, which is closely associated with the SVZ microenvironment, may attract glioma stem cells to the SVZ and promote their invasiveness.^8^ Furthermore, GBM with SVZ contact exhibits a distinct transcriptomic profile, with significant upregulation of Notch pathway-related genes, such as HES4 and DLL3, which are associated with poor survival.^9^ Additionally, basic studies have indicated that NSCs in the SVZ contribute to GBM development in distant brain regions and promote tumor proliferation, migration, and tumorigenicity, suggesting that SVZ is closely related to the origin and progression of glioblastoma.^10,11^ In clinical research, GBM with SVZ contact at initial diagnosis has a poorer prognosis and tends to exhibit distinct MRI features.^12–15^ In this study, we aim to investigate the association between MRI features and SVZ contact in IDH-wild-type GBM, as well as their prognostic significance to guide personalized diagnosis and treatment.

## Patients and methods

### Patient cohort

The imaging and clinical data of 371 patients with IDH-wild-type GBM were obtained from The Cancer Imaging Archive (TCIA) (https://www.cancerimagingarchive.net/). The patient inclusion criteria were as follows: (1) According to the latest 2021 World Health Organization (WHO) classification of gliomas^16^, patients were confirmed as IDH-wild-type GBM, a supratentorial tumor. (2) MRI scans included T1-weighted contrast-enhanced (T1C) images and T2-weighted (T2) images preoperatively. (3) Patients with preoperative lesion exhibiting contrast enhancement on T1C image. (4) patients aged ≥ 18 years. The exclusion criteria were patients with incomplete clinical data, poor image quality, and a history of biopsy or surgery for a brain tumor. This retrospective study was approved by the Ethics Committee of the First Affiliated Hospital of Chongqing Medical University (approval no. 2019–178). The requirement for informed consent was waived, as all patient data were obtained from TCIA, which provides fully de-identified and publicly available datasets. The study followed the principles of the Declaration of Helsinki.

### MRI protocol

The preoperative T1C and T2 images of TCIA were acquired using 1.5T and 3.0T scanners from Siemens (TrioTim, Verio, Aera, Avanto, Magnetom Vida, Skyra, Espree, Symphony Tim, Trio) and GE (Discovery MR750w, Optima MR450w). The parameters for T1C sequence were as follows: repetition time (TR)/echo time (TE), 5.536–2200 ms/1.336–20 ms, and slice thickness, 1 mm. The parameters for T2 sequence were as follows: TR/TE, 900–6550 ms/65.664–458 ms, and slice thickness, 0.9–5 mm. The matrix size of MRI sequences was either 256×256 or 512×512.

### MR imaging features

**FIGURE 1.**
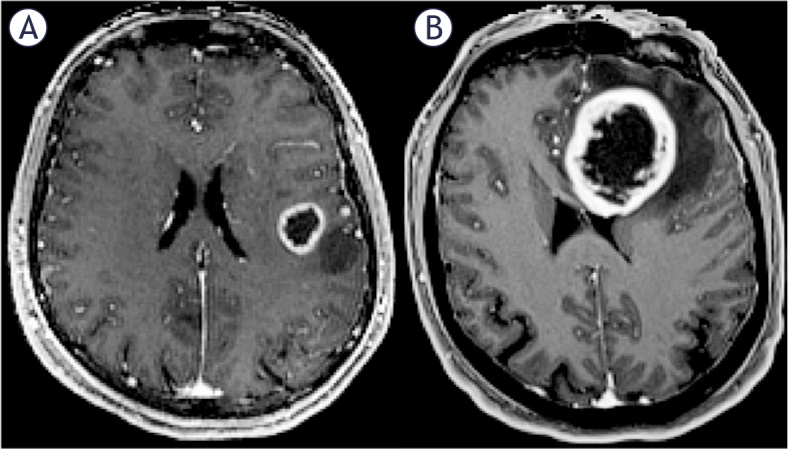
The anatomical relationship between glioblastoma and the subventricular zone (SVZ). **(A)** Contrast-enhancing tumor lesions that do not contact the SVZ are classified as the SVZ non-contact group. **(B)** Contrastenhancing tumor lesions that contact the SVZ are classified as the SVZ contact group.

**FIGURE 2.**
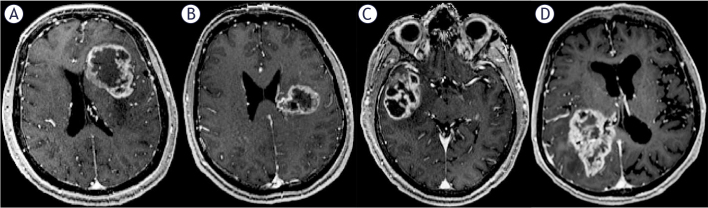
Glioblastomas with SVZ contact are further divided into four groups according to the specific regions of the lateral ventricle contacted. **(A)** Tumor contacting the frontal horn of the left lateral ventricle. **(B)** Tumor contacting the body of the left lateral ventricle. **(C)** Tumor contacting the temporal horn of the right lateral ventricle. **(D)** Tumor contacting the occipital horn of the right lateral ventricle.

**FIGURE 3.**
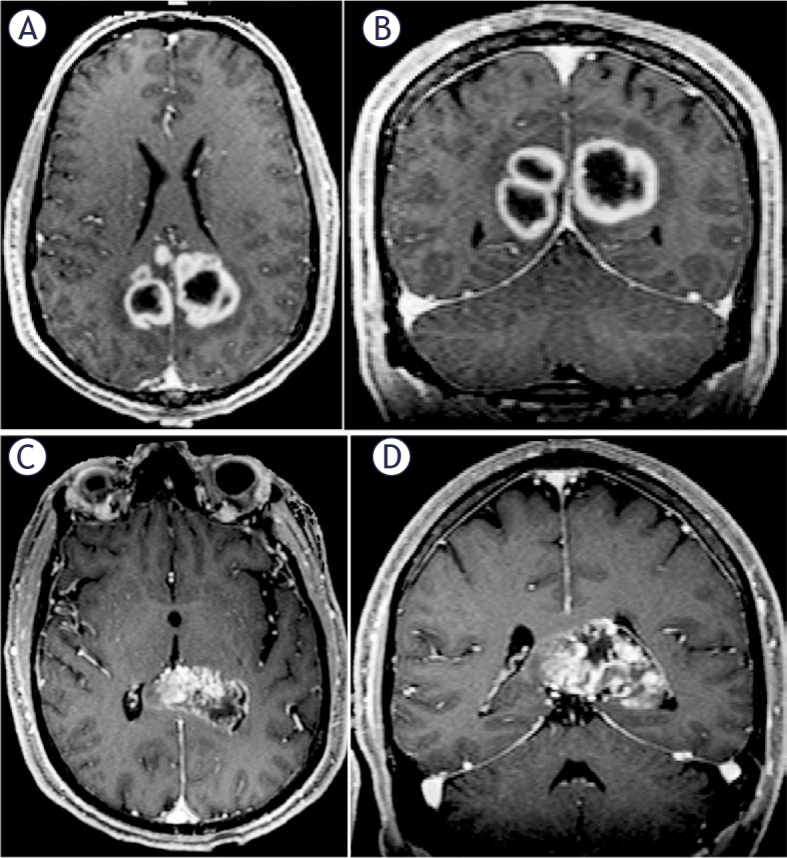
Axial **(A)** and coronal **(B)** contrast-enhanced T1-weighted MR images of a GBM patient with multifocal manifestations. Panels **(C)** and **(D)** illustrate another GBM patient with a tumor crossing the midline, where the contrast-enhanced lesion extends from the corpus callosum to the contralateral hemisphere.

The contact between the contrast-enhancing portion of the tumor and the SVZ was evaluated using preoperative T1C MPR images. In this study, SVZ contact was defined as the overlap between the tumor enhancement region and the ventricular region, all patients were divided into SVZ contact group and SVZ non-contact group (Figure 1). Referring to lateral ventricle partitions^17^, the SVZ contact group was further divided into the frontal horn (+), body (+), temporal horn (+), and occipital horn (+) subgroups based on different contact region (Figure 2). For tumors that contacted multiple parts of the lateral ventricle simultaneously, the region with the largest area of contact with the contrast-enhancing portion of the tumor on T1C MPR images was designated as the primary contact area. The three-dimensional maximum diameter (cm) on T1C images was used to assess tumor size. Peritumoral edema was defined as a region of high T2 signal intensity surrounding the tumor, measured by the maximum distance from the tumor margin to the outer edge of the edema on T2 images. Necrosis was characterized by irregular peripheral enhancement with a nonuniform wall and central high T2 signal intensity, lower than that of CSF. Cystic lesions were defined by peripheral enhancement with a thin, uniform wall and central high T2 signal intensity, similar to that of CSF. Significant enhancement was defined as enhancement with a signal intensity equal to or greater than that of the cavernous sinus. Multifocal lesions were defined as the presence of more than one contrast-enhancing lesion (Figure 3 A,B). Tumor crossing the midline was identified by the presence of abnormal signal extending across the midline on T1C images (Figure 3 C,D). The above imaging features of GBM were assessed by two neuroradiologists who were blinded to the patient’s clinical information using the open-source software ITK-SNAP 4.0 (http://www.itksnap.org/). If there were discrepancies between the two neuroradiologists, they were resolved by consensus.

### Statistical analysis

In this study, all statistical analyses were performed using SPSS software (version 27.0). Continuous variables were first tested for normality. Normally distributed variables were presented as the mean ± standard deviation and compared using Student’s t-test. Non-normally distributed variables were expressed as the median or quartile M (P25, P75) and compared between groups using the Mann-Whitney U test. Categorical variables were reported as frequencies and percentages (n, %). The chi-square test was used for comparisons of categorical variables, while Fisher’s exact test was applied when the expected frequency was < 5. Kaplan-Meier survival analysis was employed to assess differences in survival between groups. Cox proportional hazards regression models were used to evaluate the prognostic significance of clinical and imaging features. For all statistical analyses, a two-tailed p-value < 0.05 was considered to indicate statistical significance.

## Results

A total of 371 patients with IDH-wild-type glioblastoma were included in this study. Based on the analysis of the relationship between the tumor and the subventricular zone on preoperative T1C images, 239 patients (64.4%) were classified into the SVZ contact group, while 132 patients (35.6%) were classified into the SVZ non-contact group. Among the 239 patients in the SVZ contact group, 51 patients (21.3%) had frontal horn contact, 26 patients (10.9%) had body contact, 62 patients (26.0%) had temporal horn contact, and 100 patients (41.8%) had occipital horn contact. There were no significant differences between the two groups in terms of gender, age, peritumoral edema, enhancement degree, and multifocal lesions. However, significant differences were observed in OS, tumor size, tumor shape, enhancement type, cystic lesion, necrosis, and crossing the midline (Table 1).

**TABLE 1.** Comparison of clinical and MRI characteristics between the subventricular zone contact and non-contact groups

Characteristic	SVZ Contact (n = 239)	SVZ Non-contact (n = 132)	p value
Age (year)	65 ± 11	63 ± 10	0.085
Gender, n (%)			0.237
Male	149 (62.3)	74 (56.1)	
Female	90 (37.7)	58 (43.9)	
OS (month)	11.0 (5.0–18.0)	17.5 (12.0–28.5)	< 0.001^*^
Tumor size (cm)	5.07 ± 1.35	3.31 ± 1.12	< 0.001^*^
Peritumoral edema (cm)	1.83 (1.02–2.70)	1.70 (0.55–2.62)	0.192
Tumor shape/n (%)			0.016^*^
Regularity	10 (4.2)	14 (10.6)	
Irregularity	229 (95.8)	118 (89.4)	
Enhancement type/n (%)			0.030^*^
Ring	15 (6.2)	17 (12.9)	
Non-ring	224 (93.8)	115 (87.1)	
Enhancement degree/n (%)			0.386
Significant	212 (88.7)	113 (85.6)	
Non-significant	27 (11.3)	19 (14.4)	
Cystic lesion/n (%)			0.004^*^
Yes	83 (34.7)	27 (20.5)	
No	156 (65.3)	105 (79.5)	
Necrosis/n (%)			0.011^*^
Yes	237 (99.2)	125 (94.7)	
No	2 (0.8)	7 (5.3)	
Multifocal lesions (%)			0.050
Yes	37 (15.5)	11 (8.3)	
No	202 (84.5)	121 (91.7)	
Crossing the midline (%)			< 0.001^*^
Yes	28 (11.7)	0 (0.0)	
No	211 (88.3)	132 (100.0)	

1 OS = overall survival; SVZ = subventricular zone;

* indicates p < 0.05

**FIGURE 4.**
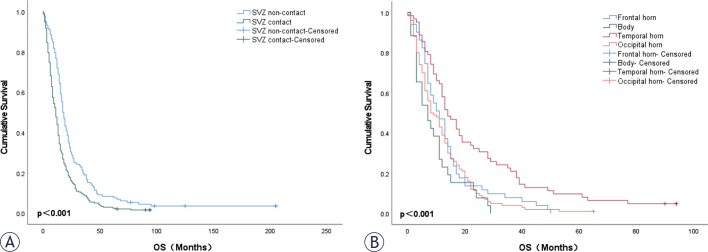
Kaplan-Meier curves for overall survival in glioblastoma: **(A)** Patients with and without SVZ contact; **(B)** Patients with tumors contacting specific SVZ regions. SVZ =subventricular zone

Compared to the SVZ non-contact group, patients in the SVZ contact group had significantly shorter overall survival (11.0 *vs*. 17.5 months, p < 0.001), larger tumor size (5.07 *vs*. 3.31 cm, p < 0.001), a higher likelihood of crossing the midline (11.7% *vs*. 0%, p < 0.001), and were more prone to cystic lesion and necrosis. Univariate Cox regression analysis identified age, tumor size, enhancement type, multifocal lesions, crossing the midline, and SVZ contact as unfavorable prognostic factors for patients with IDH wild-type glioblastoma. Multivariate Cox regression analysis further revealed that SVZ contact (p = 0.027, HR = 1.364, 95% CI: 1.037–1.794), multifocal lesions (p = 0.010, HR = 1.548, 95% CI: 1.111–2.158), and age (p < 0.001, HR = 1.024, 95% CI: 1.014–1.034) remained statistically significant predictors of overall survival, indicating that these factors are independent prognostic risk factors for patients with IDH-wild-type glioblastoma (Table 2). Patients in the SVZ contact group had significantly worse overall survival compared to those in the SVZ non-contact group (p < 0.001, Figure 4A). Among patients in the SVZ contact group, the specific region of SVZ contact was associated with OS (p < 0.001, Figure 4B). Specifically, contact of the temporal horn was associated with longer OS, while contact of the body was associated with shorter OS.

**TABLE 2.** Univariate and multivariate Cox regression analyses for overall survival in glioblastoma patients

Parameter	Univariate analysis			Multivariate analysis		
	HR	95% CI	P value	HR	95% CI	P value
Age	1.025	1.015–1.036	< 0.001*	1.024	1.014–1.034	< 0.001*
Gender	1.009	0.817–1.247	0.933			
Tumor size	1.112	1.039–1.190	0.002*	1.053	0.966–1.148	0.239
Peritumoral edema	0.950	0.876–1.029	0.207			
Tumor shape	0.783	0.513–1.195	0.257			
Enhancement type	0.687	0.475–0.993	0.046*	0.771	0.532–1.117	0.170
Enhancement degree	1.004	0.732–1.377	0.980			
Cystic lesion	0.904	0.722–1.133	0.383			
Necrosis	1.008	0.519–1.956	0.982			
Multifocal lesions	1.635	1.201–2.225	0.002*	1.548	1.111–2.158	0.010*
Crossing the midline	1.763	1.196–2.599	0.004*	1.415	0.936–2.138	0.099
SVZ contact	1.650	1.326–2.052	< 0.001*	1.364	1.037–1.794	0.027*

1 CI = confidence interval; HR = hazard ratio; SVZ = subventricular zone;

* indicates p < 0.05, the p value is statistically significant

## Discussion

In our study, among the 371 glioblastoma patients, 239 cases (approximately 64%) exhibited subventricular zone contact on MRI, consistent with previous observations indicating that 60%–70% of newly diagnosed GBM patients show SVZ contact.^13^ We analyzed the clinical and imaging characteristics of IDH-wild-type GBM patients with SVZ contact and further confirmed that SVZ contact serves as an independent negative prognostic factor for GBM patients. In addition, age, tumor size, multifocal lesions, and crossing the midline were also identified as prognostic risk factors. Compared to the SVZ non-contact group, patients in the SVZ contact group exhibited shorter overall survival, larger tumor size, higher proportions of crossing the midline and multifocal lesions, as well as an increased tendency for cystic lesion and necrosis. These findings suggest that SVZ contact not only influences the biological behavior of the tumor but is also closely associated with clinical prognosis.

In the analysis of survival and prognosis, we identified SVZ contact as an independent prognostic factor in patients with IDH-wild-type glioblastoma. This finding is consistent with a previous meta-analysis^18^, and supported by clinical studies. For instance, Hallaert *et al*.^13^ analyzed 214 glioblastoma patients and found that those with SVZ contact had shorter overall survival and progressionfree survival. Additionally, a study by Najbauer *et al*.^19^ further supported SVZ contact as a significant indicator of poor prognosis and investigated the relationship between molecular markers in the SVZ microenvironment and patient outcomes through genomic sequencing. Our study demonstrated a significant correlation between the specific SVZ contact region and OS, with patients exhibiting temporal horn contact having a relatively longer OS, whereas those with body contact having a shorter OS. However, previous studies have reported inconsistent findings. Some studies have suggested that occipital horn contact is associated with poorer prognosis^20,21^, while others found no significant differences in OS among different SVZ contact regions.^15,22^ These discrepancies may be attributed to heterogeneity in study design, subjective criteria for SVZ subregion classification, and variations in the biological characteristics of the SVZ microenvironment. Previous studies have indicated that different SVZ regions exhibit distinct cellular compositions and molecular characteristics^23,24^, which may lead to tumor contacting specific regions exhibiting distinct biological behaviors and invasion patterns, ultimately affecting patient prognosis. Moreover, the current classification of SVZ regions is primarily based on anatomical subdivisions, which may not fully reflect regional molecular and microenvironmental differences. Although our study provides new insights into the prognostic significance of SVZ contact, the underlying molecular mechanisms remain unclear. Future research should focus on large-scale prospective studies, single-cell sequencing, spatial transcriptomics, and radiomics to further elucidate the molecular characteristics and microenvironmental dynamics of different SVZ contact regions, thereby uncovering their potential associations with tumor invasion and prognosis. SVZ contact may increase the surgical complexity and postoperative complication risk in glioblastoma patients. Mistry *et al*.^25^ demonstrated that SVZ contact was associated with an increased risk of post-treatment hydrocephalus and leptomeningeal dissemination. Additionally, another study reported that glioblastoma with SVZ contact was more likely to disseminate along the cerebrospinal fluid pathways, and patients with intraoperative ventricular entry had a higher risk of postoperative leptomeningeal dissemination than those without ventricular entry.^26^ Therefore, to reduce postoperative complications, neurosurgeons are advised to minimize ventricular entry during surgery. However, the impact of ventricular opening on prognosis remains controversial. Saito *et al*.^27^ found that a greater extent of ventricular opening was associated with longer survival, possibly due to the removal of a larger proportion of tumor stem cells residing within the SVZ. Nevertheless, surgical trauma may potentially affect neurological function, thereby reducing patients’ quality of life and overall survival. In summary, GBM with SVZ contact is often associated with poor prognosis and poses technical challenges for neurosurgical resection. Future research should focus on optimizing the balance between maximal tumor resection and minimizing postoperative complications to improve patient outcomes.

In this study, we observed that GBM with SVZ contact exhibited larger tumor size, consistent with previous studies.^13,19,28,29^ This characteristic may be attributed to the potential malignant transformation, strong proliferative and migratory capacities of NSCs within the SVZ microenvironment. Additionally, the SVZ is in extensive contact with cerebrospinal fluid, which can enrich various growth factors and promote the proliferation of cancer cells. We also observed that multifocal lesions were more frequently present in the SVZ contact group, concordant with findings from previous studies.^15,30,31^ Lim *et al*.^30^ were the first to report that GBM with SVZ contact was more likely to exhibit multifocal lesions and distant recurrence. Gupta *et al*.^32^ revealed that GBM with SVZ contact displayed distinct tangential, radial, or multipolar migratory patterns, which may explain the higher incidence of multifocal lesions in these tumors. Additionally, these tumors more frequently exhibit a butterflylike growth pattern across the midline, suggesting corpus callosum invasion. This invasion disrupts the barrier function of the corpus callosum’s dense white matter tracts against interstitial edema, tumor spread, and inflammatory infiltration.^33^ These imaging characteristics indicate that GBM with SVZ exhibits greater invasiveness, proliferation, and biological heterogeneity. These tumors are often located in anatomically complex deep brain regions and frequently exhibit multifocal characteristics, increasing surgical difficulty of complete tumor resection, often leading to poorer outcomes. Therefore, SVZ contact not only indicates a more aggressive biological behavior of the tumor but also poses challenges for clinical treatment, warranting further investigation and attention.

We also observed a higher incidence of cystic lesion in GBM with SVZ contact. However, Zhao *et al*.^28^ reported a lower incidence of cystic lesion in these tumors. It has been reported that the formation of intratumoral cystic degeneration in gliomas is primarily associated with the disruption of the blood-brain barrier, leading to the exudation and accumulation of plasma proteins.^34^ Additionally, factors such as tumor liquefactive necrosis and hemorrhagic liquefaction may also contribute to cyst formation. Regarding the prognostic significance of cystic degeneration in gliomas, Park *et al*.^35^ conducted a retrospective analysis of preoperative MRI images from 158 patients with low-grade gliomas and found that tumor cystic degeneration might be a favorable prognostic factor. However, another study suggested that cystic degeneration are not an independent prognostic factor for lowgrade gliomas.^36^ For GBM, Zhou *et al*.^37^ analyzed imaging characteristics from 180 patients and observed that cystic degeneration may be associated with longer survival. Pathological studies have indicated that cystic GBM exhibits less peritumoral edema, clearer boundaries, and fewer infiltrating tumor cells, which may lead to reduced residual tumor burden postoperatively and potentially better prognosis.^38^ However, a large cohort study did not confirm a significant correlation between cystic degeneration and GBM prognosis.^39^ In this study, although there was a difference in the incidence of cystic lesion between the SVZ contact and non-contact groups, no significant association was identified between cystic lesion and overall survival. Therefore, the prognostic impact of cystic degeneration requires further validation through larger sample sizes and multicenter studies.

This study has several limitations. First, as a single-center retrospective study, it is inevitably subject to selection bias. Second, due to limited data availability, this study did not include other potential prognostic factors associated with glioblastoma, such as KPS scores, MGMT status, and extent of resection. Third, the tumor margins and SVZ contact were assessed solely using T1C images, which may not accurately reflect the true tumor boundaries of glioblastoma. Therefore, incorporating multiparametric imaging analysis is critical for a more comprehensive assessment. Fourth, the interpretation of SVZ subregions and tumor contact remains somewhat subjective. Future studies could integrate microenvironmental characteristics and utilize automated segmentation methods to more objectively identify SVZ contact, thereby reducing interpretation bias and minimizing experimental error. Future studies should include larger multicenter cohorts and combine multiparametric imaging analyses with clinical and molecular characteristics to further validate our findings.

## Conclusions

Our study demonstrates that SVZ contact is an independent prognostic factor for overall survival in IDH-wild-type glioblastoma patients. GBM with SVZ contact is typically characterized by larger tumor size, a higher proportion of crossing the midline and multifocal lesions, suggesting that these tumors may exhibit increased aggressiveness, proliferative and migratory capabilities. However, further studies are needed to verify whether differences in cystic degeneration and necrosis exist between SVZ contact and SVZ non-contact groups, and to elucidate their prognostic significance. Early and systematic evaluation of tumor imaging characteristics can provide valuable insights for clinical neurosurgeons, enabling better prediction of patient outcomes and the development of individualized treatment strategies. Moreover, strategies targeting NSCs in the SVZ region and eradicating tumor-initiating cells through radiotherapy could help suppress tumor progression at its source, potentially prolonging patient survival. In the future, the SVZ may emerge as a critical target for radiotherapy and other targeted therapies in glioblastoma treatment.
